# Metastatic mastery: the uncommon journey of papillary thyroid carcinoma to the brain (a case report)

**DOI:** 10.11604/pamj.2023.45.20.40017

**Published:** 2023-05-05

**Authors:** Deeksha Rana, Samarth Shukla, Preeti Mishra, Priyal Shrivastava, Pratapsingh Parihar

**Affiliations:** 1 Department of Pathology, Jawaharlal Nehru Medical College, Sawangi (Meghe), Wardha, Maharashtra, India,; 2 Department of Radiodiagnosis, Jawaharlal Nehru Medical College, Sawangi (Meghe), Wardha, Maharashtra, India

**Keywords:** Thyroid malignancy, papillary thyroid carcinoma, brain metastases, case report

## Abstract

Papillary thyroid carcinoma is one of the most common thyroid malignancy, often has excellent prognosis and low incidence of distant metastatic conditions. Brain metastases from papillary thyroid carcinoma has a rare occurrence, with patients presenting with non-specific symptoms such as headaches, cognitive changes etc., and poor survival outcomes. The standard protocol for diagnosis and treatment remains controversial. We report a patient who presented with cerebral metastasis prior to the diagnosis of papillary thyroid carcinoma, review the current literature, and explain our approach on the basis of clinical, pathological, and radiological data. A 60-year-old hypertensive male presented with lower back pain, bilateral lower limb weakness, occasional episodes of frontal headache and personality changes. The diagnostic evaluation included computed tomography (CT) scan, magnetic resonance imaging (MRI) with and without contrast enhancement, and color Doppler. Intra-axial complex solid cystic mass lesion in the right parieto-occipital region with significant perilesional oedema, and imaging characteristic of neoplastic etiology were observed. He was posted for excision of tumor and underwent right occipital craniotomy. Histopathological analysis of the surgical specimen confirmed papillary carcinoma of the thyroid gland. Brain metastases from thyroid malignancy is a sign of detrimental prognosis, hence, thorough clinical, radiological and pathological evaluation for rapid detection is critical. Neurosurgical removal along with radiotherapy should be considered as treatment of choice. The information obtained contributes towards better management and overall long-term outcomes.

## Introduction

Thyroid malignancies are accounted as one of the most common endocrine malignancies worldwide [1], with papillary thyroid carcinoma (PTC) being the predominant form as its prevalence contributes to 80% - 93% in comparison to all other differentiated and undifferentiated thyroid malignancies [2]. Conditions such as Hashimoto thyroiditis, ionizing radiation during adolescence, and familial incidence of approximately 4.5% are some of the reported risk factors for papillary thyroid carcinoma [2]. It primarily occurs in middle age, has female predominance with a ratio of 3: 1, mainly affects the thyroid gland proper, thyroglossal duct, lingual thyroid, and other ectopic thyroid tissue with features of epithelial origin with follicular differentiation, commonly less severe clinical course with overall favourable prognosis making it a distinct characteristic condition [3]. On the other hand, brain metastases (BMs) have been regarded as the commonest neurological complication in cancer contributing to substantial morbidity and mortality cases. The clinical presentation seen in patients is often headaches, seizures, cognitive changes, focal deficits etc. associated with growing mass lesions and significant edema [1]. Brain metastases typically occur in cases of lung carcinoma, melanomas, renal carcinomas, breast carcinomas, and colorectal malignancies reported to be 20%, 6.9%, 6.5%, 5.1%, and 1.8% respectively [4]. The diagnosis made is mostly based on radiology and pathological data, and its management generally comprises surgery, radiosurgery, and conventional radiotherapy, used alone or in combination [1].

In recent years, we have witnessed an increase in papillary thyroid carcinoma cases, often termed as thyroid cancer epidemics, however, with a very low incidence of distant metastasis [5]. Papillary thyroid carcinoma metastases commonly spread to regional cervical lymph nodes which have proven to be cases of low-risk tumour, with excellent prognoses with 5-year and 10-year survival rates equivalent to 96% and 93% respectively [2]. However, brain metastases from primary thyroid cancer have been recognized as a rare occurrence with an incidence of 0.1-5% and reported cases are shown to be aggressive with a bad prognosis of less than a year life [5,6]. There is no proper approach towards the management of such distant metastatic cases because of their less occurrence and consequently, decisions are mainly based on retrospective studies, case reports, standard care for brain metastases from other types of malignancies, etc. Thus, there is a need for early detection and appropriate treatment for good outcomes [7].

We report one rare case of a 60-year-old male patient with clinical details of histologically proven brain metastases in the occipital lobe from undiagnosed primary papillary thyroid carcinoma, with initial presentation of neurological symptoms and explain our approach on the basis of clinical, radiological, and pathological findings of this unanticipated clinical presentation.

## Patient and observation

**Patient information:** a 60-year-old male patient was brought by his relatives to the hospital and presented with chief complaints of lower back pain followed by bilateral lower limb weakness for six months associated with generalized weakness for one month, along with history of occasional episodes of frontal headache and personality changes claimed by his relatives. The patient had no significant family history.

**Clinical findings:** neurological examination revealed left-sided hemiparesis and impairment in his recent and remote memory as well as judgment and emotional functions. On admission, the patient was a known case of hypertension for 2 years, and on medication, and has no history of diabetes mellitus.

**Timeline:** patient was admitted to Neuro-Intensive Care Unit (ICU). Complete blood count and biochemical parameters were within normal limits.

**Figure 1:**
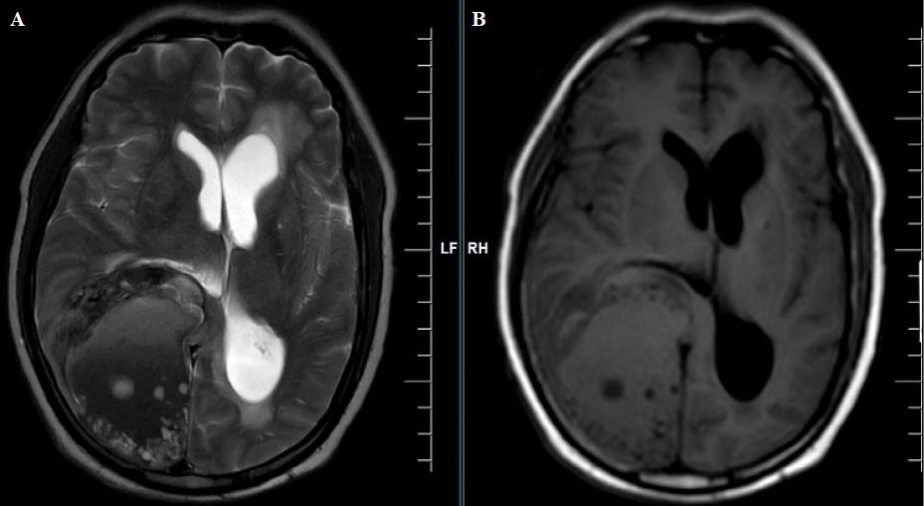
A,B) MRI of the brain axial section, lesion in right occipital lobe appearing hypointense on T1-weighted image (T1WI) and hyperintense on T2-weighted image (T2WI) with midline shift to left

**Figure 2:**
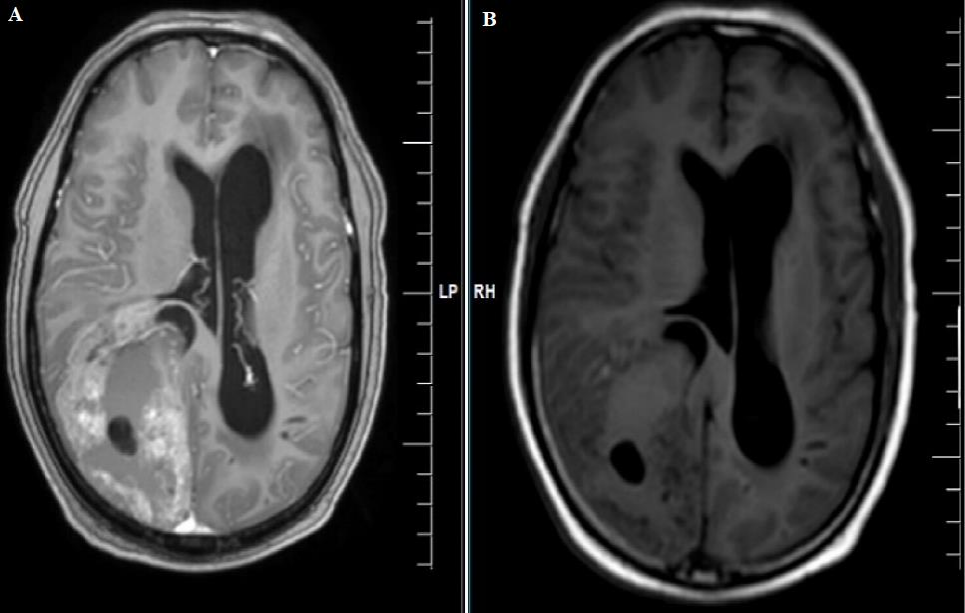
A,B) MRI of the brain axial section showing post-contrast enhancement in right occipital lobe

**Figure 3:**
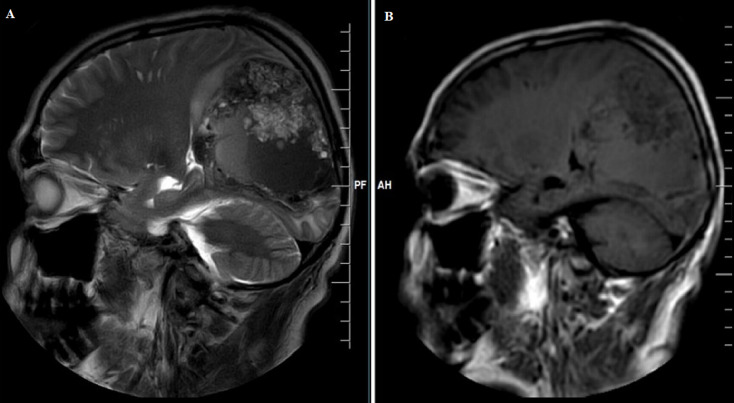
A,B) MRI of the brain showing multiple areas of blooming in right parieto-occipital region on susceptibility weighted imaging (SWI)

**Figure 4:**
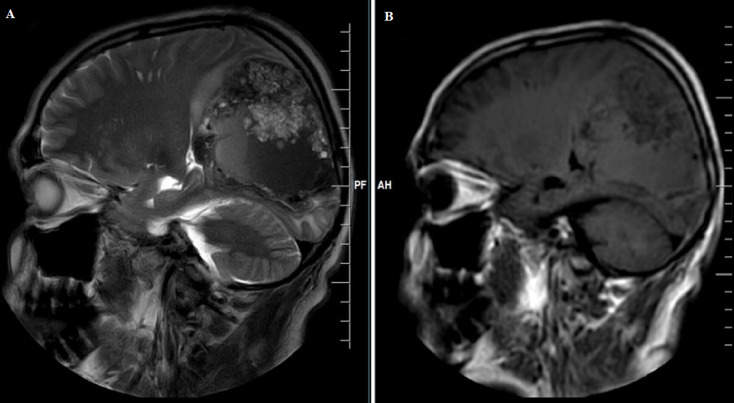
A,B) MRI of the brain sagittal section, lesion in parieto-occipital area

**Diagnostic assessment:** magnetic resonance imaging (MRI) brain contrast revealed an intra-axial complex solid cystic mass lesion measuring 7.9x5.6x7.5 cms (APXTRANSXCC) in the right parieto-occipital parafalcine region with blood fluid level, significant perilesional oedema, mass effect and imaging characteristic of neoplastic etiology (Figure 1, Figure 2, Figure 3, Figure 4).

**Diagnosis:** through physical examination and radiological investigations, the patient was diagnosed with a right occipital lobe tumor (para sagittal tumor) and was posted for excision of the tumor.

**Figure 5:**
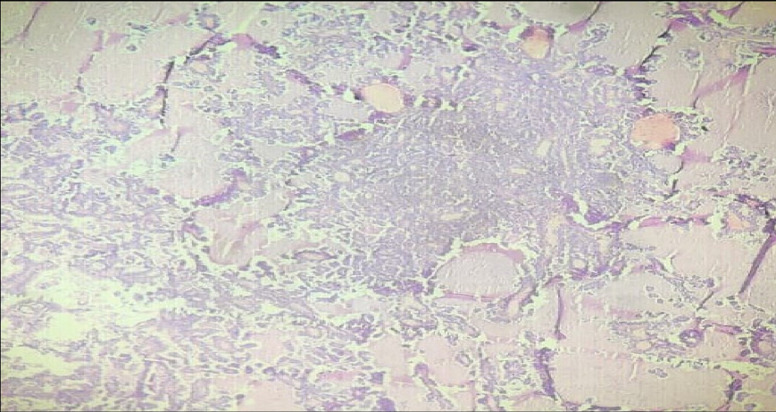
under 10X microscopy, H&E section showing metastatic deposits of papillary thyroid carcinoma

**Figure 6:**
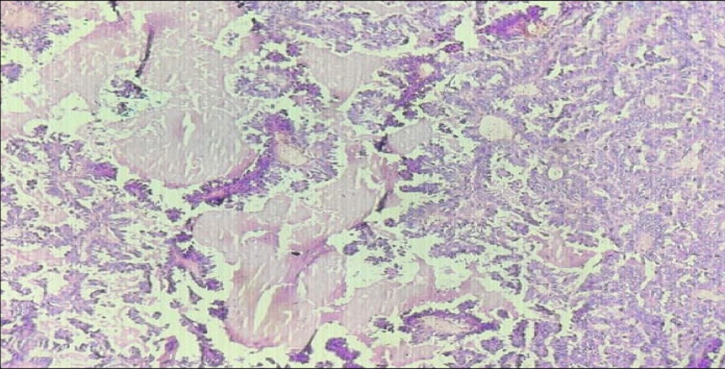
in 20X microscopy showing papillae, papillary arrangement lined by hyperchromatic tumour cells

**Figure 7:**
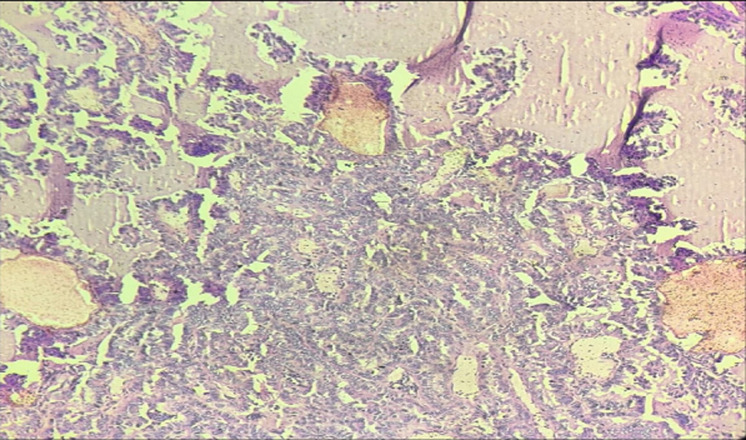
in 40X microscopy, brain parenchyma showing infiltration by acute inflammatory cells, mainly neutrophils along with necrosis and few areas of haemorrhage; these papillary structures showing nuclear groove, nuclear opacities

**Therapeutic interventions:** pre-anesthetic checkup, medicine and cardiac fitness, and high-risk consent were taken for surgery. He underwent right occipital craniotomy; intraoperatively, a well-capsulated minimally vascular lesion was found and excised completely. Grossly, the specimen was measuring approximately 11 x 5 x 3 cms, and was reddish brown, solid, and firm. The section was taken for histopathology examination in the department of pathology and microscopy revealed metastatic deposits of papillary carcinoma of the thyroid (Figure 5, Figure 6, Figure 7).

**Follow-up and outcome of interventions:** post-operatively, the patient was hemodynamically and vitally stable, maintaining saturation at 100%, and was extubated.

**Patient perspective:** the patient and his relatives expressed gratitude for the care and treatment received and expressed optimism about his progress.

**Informed consent:** the patient and his relatives gave written informed consent for the publication of this case report. The study involving radiological and histopathological images was de-identified to protect patient´s privacy and maintain confidentiality.

## Discussion

Papillary thyroid carcinoma is defined through distinctive nuclear features such as enlarged, elongated and glassy nuclei with irregular nuclear contour and nuclear pseudoinclusions [8]. This cancer is regarded as the most common of all thyroid malignancies with prevalence rate of 80-90%, however, with excellent prognosis and low incidence of distant metastatic spread to lungs, followed by bone, and uncommonly to sites like skin, liver, and brain [2,5]. On the basis of moderately few large retrospective studies, the most commonly reported prevalence of brain metastases from papillary thyroid carcinoma is around 1%, however, the actual prevalence is believed to be much high because, as the cases of thyroid cancer are increasing; so is the diagnosis of metastatic cases annually [9]. McWilliams *et al*. mentions a study conducted by Dinneen *et al*. who reported that around 18% of patients developed brain metastases from papillary thyroid carcinoma, and the brain was the second most common site for metastases [7]. A study was conducted by Chiu AC *et al*. where about 47 cases of brain metastases from thyroid cancer were reviewed and reports noted that brain metastases were a primary clinical feature at initial presentation in 15% of the cases, were identified during the subsequent course of the disease in 68%, and were only discovered at autopsy in 23%. There was evidence of aggressive clinical courses in older patients. Also, after diagnosis, disease-specific mortality was 78%, with a median product-limit survival of 4.7 months [10].

The case report presented is singular and rare because of its unusual clinical manifestations, i.e. initial neurological findings, and uncommon diagnosis, i.e. diagnosis made after histopathological investigation of the mass lesion which was possible only after surgery. To our knowledge, we found a similar instance in only one case published by Al-Dhahri *et al*. 2009 [2] who reported a case of a 75-year-old female diagnosed with haemorrhagic mass lesion in the left cerebellum, underwent left occipital craniotomy and excision of tumour showed metastases consistent with thyroid primary.

The clinical presentation of these metastatic cases is usually different and non-specific such as headaches, focal neurological dysfunction, cognitive deficits, stroke, nausea, diplopia, visual disturbances, ataxia, seizures, etc. with mass lesion present commonly in the areas of the frontal, parietal and temporal lobe, but in some cases, cerebello-pontine angle, cerebellum, pituitary gland, and cavernous sinus as unfamiliar locations are also reported [1,5]. In the case discussed, due to the presence of a mass lesion, the patient presented with complaints of occasional episodes of frontal headache and significant cognitive changes.

There is no definitive approach for these metastatic cases as the data obtained through the patient´s clinical history, physical and neurological examination, imaging exams, and biochemical assessment is quite limited, hence, it is difficult to make a diagnosis based on clinical symptoms and it is noted that only histopathological reporting provides the confirmation of diagnosis [6]. Regarding the diagnostic workup, previous studies report that the time interval from initial presentation to the diagnosis of brain metastasis usually varies from few months to 35 years [5]. Reports suggest that 131I-single-photon emission computerized tomography (SPECT)/computed tomography (CT) screening is relatively more perceptive than chest X-ray or CT scan, but, less than thyroglobulin level assessment for detecting metastases [6]. Fluorine-18- fluorodeoxyglucose (FDG)-positron emission tomography (PET)/CT is another useful tool that reveals negative results for I 131 whole body scan in patients with elevated levels of serum thyroglobulin, but has a limitation of not revealing all brain metastatic lesions. Hence, MRI is the preferred imaging modality due to its high sensitivity and specificity and the most common employed method for the detection of brain metastases [6]. In our report, the patient was sent for MRI to diagnose brain metastases.

Over decades, there has been no improvement in the treatment of brain metastases making decisions regarding management without any standard guidelines, merely based on experience. Several factors like site of lesion, tumour burden, histopathological variant, radioiodine avidity etc. make treatment variable [1]. Although many treatment modalities have been used in limited intracranial metastatic papillary thyroid carcinoma cases, the results are quite equivocal. Management approaches include conservative treatment, surgical resection and radioactive iodine, external beam radiation, gamma knife radiosurgery and radioactive iodine therapy, to name a few [5].

According to various studies, the best treatment modality is surgical resection followed by radioactive iodine therapy [2]. In cases of high-risk surgery or incomplete resection, radiosurgery or adjuvant whole-brain radiotherapy can be considered, though very few studies are available to support the statement [6]. Stereotactic radiosurgery (SRS) is regarded as the best radiotherapeutic option as it is effective in all cases of small, multiple, and deep metastases. Studies show a strong association between SRS and high local control rate with longer overall survival of more than 3 years [1]. Another attractive possibility is gamma knife radiosurgery, as it is less invasive than craniotomy, and is considered in patients with limited survival outcomes [7]. Chiu *et al*. showed the use of recombinant human thyrotropin (TSH) stimulated radioiodine uptake for intracranial metastatic treatment, but no evidence of survival benefit was found from radioiodine therapy, external beam radiotherapy, or chemotherapy [10]. Wu *et al*. reported in their study that blood-brain barrier and meninges protect brain tissue from the accumulation of chemotherapeutic drugs in tumor lesions and high-dose radiation, thus, weakening the effect [9].

It is noted that a combined treatment approach is essential and management should be constantly tailored according to the individual response in each case. Ha *et al*. in his case report mentions a study by Sheu *et al*. which reported improved life expectancy through the combination of radioiodine with other modalities, however, adverse effects like cerebral edema and haemorrhage were also seen [1]. In a study by McWilliams *et al*. patients who suffered acute cerebral edema post radioiodine therapy, were given prophylactic glycerol for prophylaxis [7]. Emamhadi MR *et al*. mentions a study reported by Miranda *et al*. in which a case of brain metastases that were treated with a combination of surgical excision, whole-brain radiation therapy, and gamma knife radiosurgery showed 10-year survival outcome [6].

To summarize, brain metastases from papillary thyroid carcinoma show an aggressive clinical course in old patients and is a sign of detrimental prognosis with approximately 50% decrease in the 5-year survival rate. When approaching a patient with brain metastases, the possibility of spread from the primary thyroid gland should be kept in mind. Decisions made should include awareness of long-term risks and their respective management approaches.

## Conclusion

The incidence of brain metastases (BMs) secondary to papillary thyroid cancers (PTC) is a quite rare occurrence to approximately 1% with a poor prognosis and relatively short span of survival. The cases reported with a presentation of distant metastases in the brain before a primary diagnosis of papillary thyroid carcinoma are even more exceptional, as with our case. The knowledge of thyroid carcinoma in the clinical analysis of patients with neurological characteristics should be highly valued, aided with early investigation of thyroid imaging exams, rapid detection, close follow-up, and aggressive treatment. Among treatment modalities, surgery followed by radiotherapy is the treatment of choice for this specific type of metastasis to avoid the effects of the growing tumour. The adjuvant use of whole brain radiation therapy after surgery is a good alternative, though more studies are needed. This case report also serves the importance of reviewing the outcomes of such metastatic cases which would help to improve patient prognosis in the era of modern treatments.
